# The expression and significance of efferocytosis and immune checkpoint related molecules in pancancer samples and the correlation of their expression with anticancer drug sensitivity

**DOI:** 10.3389/fphar.2022.977025

**Published:** 2022-08-19

**Authors:** Lin Cheng, Bangbi Weng, Changsheng Jia, Lin Zhang, Bin Hu, Li Deng, Nan Mou, Fengjun Sun, Jing Hu

**Affiliations:** Department of Pharmacy, The First Affiliated Hospital of Army Medical University (Third Military Medical University), Chongqing, China

**Keywords:** efferocytosis, immune checkpoint-related molecules, immunotherapy, chemotherapy resistance, cancer

## Abstract

**Background:** The efferocytosis-related molecules have been considered to be correlated with the resistance to cancer chemotherapy. The aim of this study was to investigate the expression and significance of efferocytosis-related molecules in cancers and the correlation of their expression with anticancer drug sensitivity, and provide new potential targets and treatment options for cancers.

**Methods:** We investigated the differential expression of 15 efferocytosis-related molecules (Axl, Tyro3, MerTK, CX3CL1, Tim-4, BAI1, Stab2, Gas6, IDO1, Rac1, MFGE8, ICAM-1, CD47, CD31, and PD-L1) and other 12 common immune checkpoint-related molecules in tumor and normal tissues, the correlation between their expression and various clinicopathological features in 16 types of cancers using publicly available pancancer datasets in The Cancer Genome Atlas. We also analyzed the correlation of the expression of efferocytosis and immune checkpoint related molecules with 126 types of anticancer drugs sensitivity using drug-RNA-seq data.

**Results:** There is a panel of circulating molecules among the 27 molecules. Based on the results of differential expression and correlation with various clinicopathological features of efferocytosis-related molecules in cancers, we identified new potential therapeutic targets for anticancer therapy, such as Axl for kidney renal clear cell carcinoma, Tyro3 for liver hepatocellular carcinoma, and IDO1 for renal papillary cell carcinoma. Except for BAI1, CD31, and MerTK, the enhanced expressions of Axl, Tyro3, Gas6, MFGE8, Stab2, Tim-4, CX3CL1, IDO1, Rac1, and PD-L1 were associated with decreased sensitivity of the cancer cells to many anti-cancer drugs; however, for other common immune checkpoint-related molecules, only enhanced expressions of PD-1, CD28, CTLA4, and HVEM were associated with decreased sensitivity of the cancer cells to a few drugs.

**Conclusion:** The efferocytosis-related molecules were significantly associated with clinical outcomes in many types of cancers and played important roles in resistance to chemotherapy. Combination therapy targeting efferocytosis-related molecules and other immune checkpoint-related molecules is necessary to reduce resistance to chemotherapy.

## Introduction

Research on the immune escape mechanism has developed a variety of antitumor immune drugs, such as immune checkpoint inhibitors and tumor vaccines, which have provided new tumor treatment methods and brought new hope to patients with advanced metastatic tumors. Cytotoxic T lymphocyte-associated antigen 4 (CTLA-4) antibody, programmed death 1 (PD-1) antibody, and its ligand PD-L1 are among the fastest-growing and most promising drug categories. They have been used in hematological malignancies, malignant melanoma, non-small cell lung cancer (NSCLC), and advanced clear cell renal cell carcinoma (ccRCC) (Galon and Bruni, 2019). The combination of checkpoint inhibitors is currently one of the most promising treatment approaches. In a phase III clinical trial, the combination treatment of PD-1 antibody nivolumab and CTLA-4 antibody ipilimumab for advanced RCC obtained a higher objective response rate as well as overall survival (OS), and the patient’s quality of life was significantly improved (Motzer et al., 2018). Dual blockade of PD-1 and lymphocyte activation gene-3 (LAG-3) was also reported as a promising checkpoint blockade combination for RCC (Zelba et al., 2019). In advanced NSCLC patients treated with PD-1 axis blockers, elevated LAG-3 was significantly associated with insensitivity to PD-1 axis blockade and shorter progression-free survival (PFS) (Datar et al., 2019). Combined treatment with chemotherapy and immunotherapy has also shown clinical benefit, and the PD-1 antibody Keytruda combined with paclitaxel and platinum chemotherapy is a major advancement in chemotherapy and immunotherapy for metastatic squamous NSCLC (Pacheco, 2020). However, primary resistance occurs in the majority of patients and acquires adaptation of tumors to immune pressure in patients initially responding to therapy. Therefore, additional nonredundant actionable immunostimulatory targets need to be characterized.

Efferocytosis is a physiologic phagocytic clearance of apoptotic cells induced mainly by macrophages and dendritic cells, which modulates inflammatory responses and the immune environment and promotes the resolution of inflammation and wound healing. In the tumor microenvironment, tumor-associated macrophages (TAMs) are professional phagocytes. During efferocytosis, TAMs polarize toward an M2-like wound healing phenotype, promote the secretion of anti-inflammatory cytokines (e.g., TGF-β, IL-4, and IL-10), inhibit the secretion of inflammation-resolving cytokines (e.g., IL-1β, TNF-α, and IL-12), recruit FOXP3^+^ regulatory T cells, and suppress the functions of CD4^+^ and CD8^+^ effector T cells, subsequently facilitating immune escape of cancer cells, thus promoting tumor development and progression.

The process of efferocytosis includes four steps: release of “find-me” signals by dying cells, recognition of the ligands of apoptotic cells by the phagocytes, phagocyte downstream of engagement of apoptotic cells, and production of anti-inflammatory mediators (Morioka et al., 2019). CX3CL1 is a member of the chemokine family, and is a common “find-me” signal released by lymphocytes after apoptosis and attracts macrophages to the apoptotic site. Axl, Tyro3, and MERTK bind the ligand arrest-specific protein 6 (Gas6), while Tyro3 and MERTK bind the ligand Protein S. The ligands Gas6 and Protein S bind phosphatidylserine (PS), thus Gas6 and Protein S are bridging molecules between the TAM receptors and PS. PS receptors include the T-cell immunoglobulin mucin (Tim) family members (Tim-1, Tim-3, and Tim-4), brain-specific angiogenesis inhibitor 1 (BAI1), and stabilin-2 (Stab2). The milk fat globule-EGF factor 8 (MFGE8) is the bridging molecule between PS receptors on apoptotic cells and receptors on phagocytes. Ras-related C3 botulinum toxin substrate 1 (Rac1) is a modulator of the cytoskeleton, playing important roles in phagocytosis, mesenchymal-like migration, and adhesion (Bid et al., 2013). Intercellular adhesion molecule 1 (ICAM1) is a transmembrane glycoprotein in the immunoglobulin superfamily that plays an important role in cell adhesion of tumor cells and TAMs and signal transduction (Usami et al., 2013). The most widely studied ligands of “don’t-eat-me” signals are CD47 and CD31. Rac1 is a molecule that promotes apoptotic cell engulfment (Proto et al., 2018). Moreover, MerTK-driven efferocytosis also induces the expression of the PD-L1. Indoleamine-2, 3-dioxegenase (IDO) 1 is an immune regulator known for driving maternal-fetal antigen tolerance. Apoptotic and necrotic tumor cells, *via* efferocytosis and IDO1, respectively, promote tumor “homeostasis” and progression (Werfel et al., 2019).

It is generally considered that efferocytosis-related molecules and pathways are potential targets for antitumor therapy (Werfel and Cook, 2018; Zhou et al., 2020); however, their role in specific cancers has not been clearly defined. To identify which molecules to target and the most promising combination of antibodies that could induce robust clinical outcomes, precise knowledge of molecular expression and co-expression on immune cells in general and their expression correlation with various clinicopathological features, including tumor TNM classification, tumor stage, and patient OS, is essential.

In the current study, we investigated the protein-protein interaction (PPI) network of efferocytosis-related molecules (Axl, Tyro3, MerTK, CX3CL1, Tim-4, BAI-1, Stab-2, Gas6, MFGE8, Rac1, ICAM-1, CD47, CD31, IDO1, and PD-L1) and other immune checkpoint-related molecules, including B- and T-lymphocyte attenuator (BTLA), glucocorticoid-induced tumor necrosis factor receptor-related protein (GITR), herpes virus entry mediator (HVEM), LAG-3, PD-1, PD-L2, Tim-3, CD28, CD80, CD137, CD27, and CTLA-4 (Wang et al., 2019). The expression of efferocytosis-related molecules in tumor and normal tissues, their roles in immune subtype, correlation with the ESTIMATE score, stromal score, and immune score, and the correlation between their expression and OS in cancers was studied using publicly available pancancer datasets from The Cancer Genome Atlas. We also analyzed the correlations of the expressions of efferocytosis-related molecules and immune checkpoint-related molecules with anticancer drugs sensitivity to provide a reference for the combined use and sequential use of drugs in each tumor treatment.

## Materials and methods

### Molecular protein-protein interaction network analysis

The PPI network of the 15 efferocytosis-related molecules and 12 other immune checkpoint-related molecules was constructed using the STRING database v11.0 (http://www.string-db.org/). The minimum required interaction score cutoff was set as greater than or equal to 0.400.

### Study population and datasets

Patient phenotypic, survival, RNA-seq, and immune subtype data from The Cancer Genome Atlas (TCGA) were downloaded from the UCSC Xena Genomics Browser (http://xena.ucsc.edu/) on 7 July 2020. The drug sensitivity and RNA-seq data were provided by the CellMiner database v2.2 (https://discover.nci.nih.gov/cellminer/). Disease-free survival (DFS) data and graphs were obtained from the GEPIA2 website (http://gepia2.cancer-pku.cn/#index). The tumor type classification and the corresponding patient number are shown in Table 1.

**TABLE 1:** Tumor type classification and patient number.

Tumor type	Full name	Total (n)	Normal (n)	Tumor (n)
ACC	Adrenocortical carcinoma	79	0	79
BLCA	Bladder Urothelial Carcinoma	430	19	411
BRCA	Breast invasive carcinoma	1217	113	1104
CESC	Cervical squamous cell carcinoma and endocervical adenocarcinoma	309	3	306
CHOL	Cholangiocarcinoma	45	9	36
COAD	Colon adenocarcinoma	512	41	471
DLBC	Lymphoid Neoplasm Diffuse Large B-cell Lymphoma	48	0	48
ESCA	Esophageal carcinoma	173	11	162
GBM	Glioblastoma multiforme	173	5	168
HNSC	Head and Neck squamous cell carcinoma	546	44	502
KICH	Kidney Chromophobe	89	24	65
KIRC	Kidney renal clear cell carcinoma	607	72	535
KIRP	Kidney renal papillary cell carcinoma	321	32	289
LAML	Acute Myeloid Leukemia	151	0	151
LGG	Brain Lower Grade Glioma	529	0	529
LIHC	Liver hepatocellular carcinoma	424	50	374
LUAD	Lung adenocarcinoma	585	59	526
LUSC	Lung squamous cell carcinoma	550	49	501
MESO	Mesothelioma	86	0	86
OV	Ovarian serous cystadenocarcinoma	379	0	379
PAAD	Pancreatic adenocarcinoma	182	4	178
PCPG	Pheochromocytoma and Paraganglioma	186	3	183
PRAD	Prostate adenocarcinoma	551	52	499
READ	Rectum adenocarcinoma	177	10	167
SARC	Sarcoma	265	2	263
SKCM	Skin Cutaneous Melanoma	472	1	471
STAD	Stomach adenocarcinoma	407	32	375
TGCT	Testicular Germ Cell Tumors	156	0	156
THCA	Thyroid carcinoma	568	58	510
THYM	Thymoma	121	2	119
UCEC	Uterine Corpus Endometrial Carcinoma	583	35	548
UCS	Uterine Carcinosarcoma	56	0	56
UVM	Uveal Melanoma	80	0	80

### Differential expression analysis

Differential expression of molecules between normal and tumor tissues in the pancancer datasets was performed in R software using the ggpubr package, and *p*-values were computed with the Wilcoxon sign-rank test when normal tissue counts were greater than or equal to 10.

### Correlation with the microenvironment and immune subtype

To compute the immune and stromal cell content within the tumor microenvironment, scores were calculated using the estimate and limma packages. The corrplot package was used to assess the association of molecules with immune and stromal scores. The correlation between molecules and immune subtype was estimated with the limma, ggplot2 and reshape2 packages. The significance of the differences among immune subtypes was determined by the Kruskal-Wallis test.

### Correlation with overall survival

For OS analysis, we used the Kaplan-Meier method to draw survival curves with the survival and survminer packages.

### Correlation between drug sensitivity and RNA-seq analysis

Drug sensitivity and RNA-seq data analyses were implemented with R, including the limma, impute, ggplot2 and ggpubr packages. Correlation tests were computed with Pearson’s correlation test. Based on the drug-RNA-seq correlation, Cytoscape v3.8.0 (http://www.cytoscape.org/) was used to visualize the network.

## Results

### Protein-protein interaction network of the molecules

**FIGURE 1:**
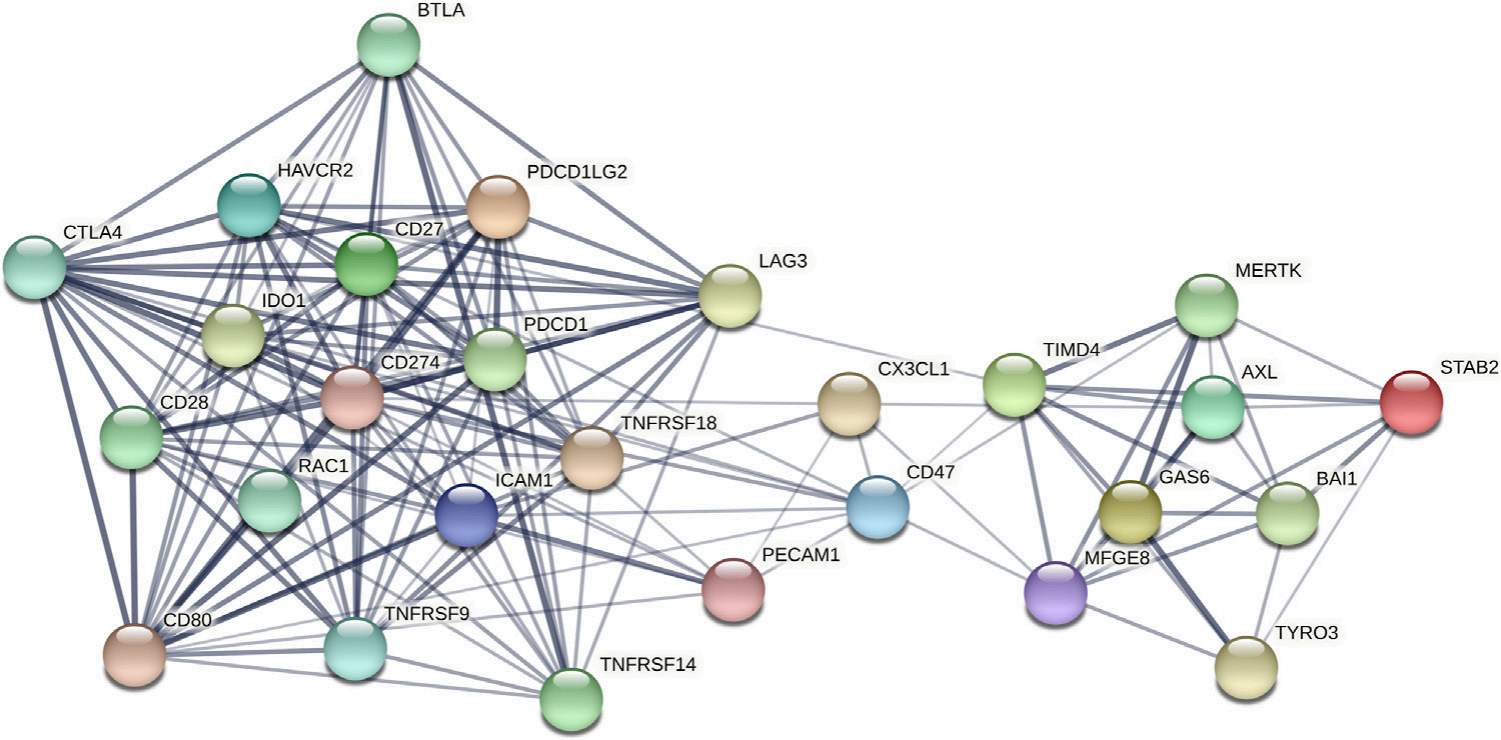
The PPI network of the 15 efferocytosis-related molecules and 12 other immune checkpoint-related molecules with confidence >0.4. The thickness of the line indicates the size of the correlation coefficient.

There is a panel of circulating molecules among the 27 molecules (Figure 1), which highlights the importance of the combination therapy of efferocytosis-related molecules and other immune checkpoint-related molecules. With an interaction score greater than or equal to 0.900, CTLA4 was strongly associated with PD-L1 (CD274), CD80, and CD28; CD80 was strongly associated with CD28, PD1, PD-L2, and ICAM-1; PD-L1 was strongly associated with CD28, CD80, PD1, and PD-L2; LAG3 was strongly associated with Tim-3 (HAVCR2); MerTK was strongly associated with Tim-4; and Gas6 was strongly associated with Axl, MerTK, and Tyro3.

### Expression signature in pancancer samples and correlation with overall survival

**FIGURE 2:**
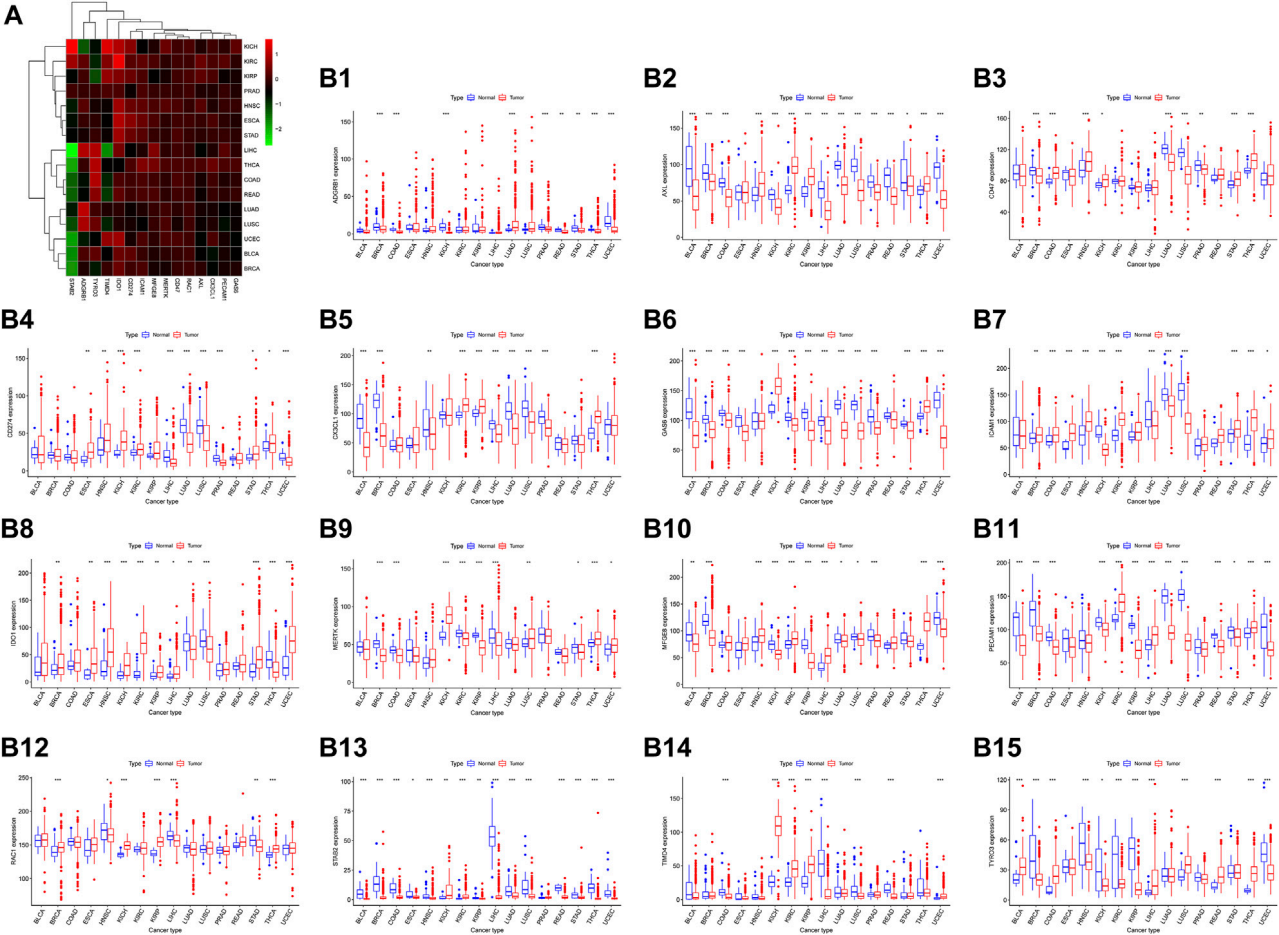
The expression level of 15 efferocytosis-related molecules in 16 types of cancers. **(A)**. Heatmap and supervised clustering of efferocytosis-related molecules with changed expression in 16 tumor tissues compared to that in normal tissues. **(B1-B15)**. The changed expression of ADGRB1 (BAI1), Axl, CD47, CD274 (PD-L1), CX3CL1, Gas6, ICAM1, IDO1, MerTK, MFGE8, PECAM1 (CD31), RAC1, STAB2, TIMD4 (Tim-4), and Tyro3 in 16 tumor tissues compared to that in normal tissues. *p < 0.05, **p < 0.01, ***p < 0.001 as assessed by the Wilcoxon signed-rank test.

**FIGURE 3:**
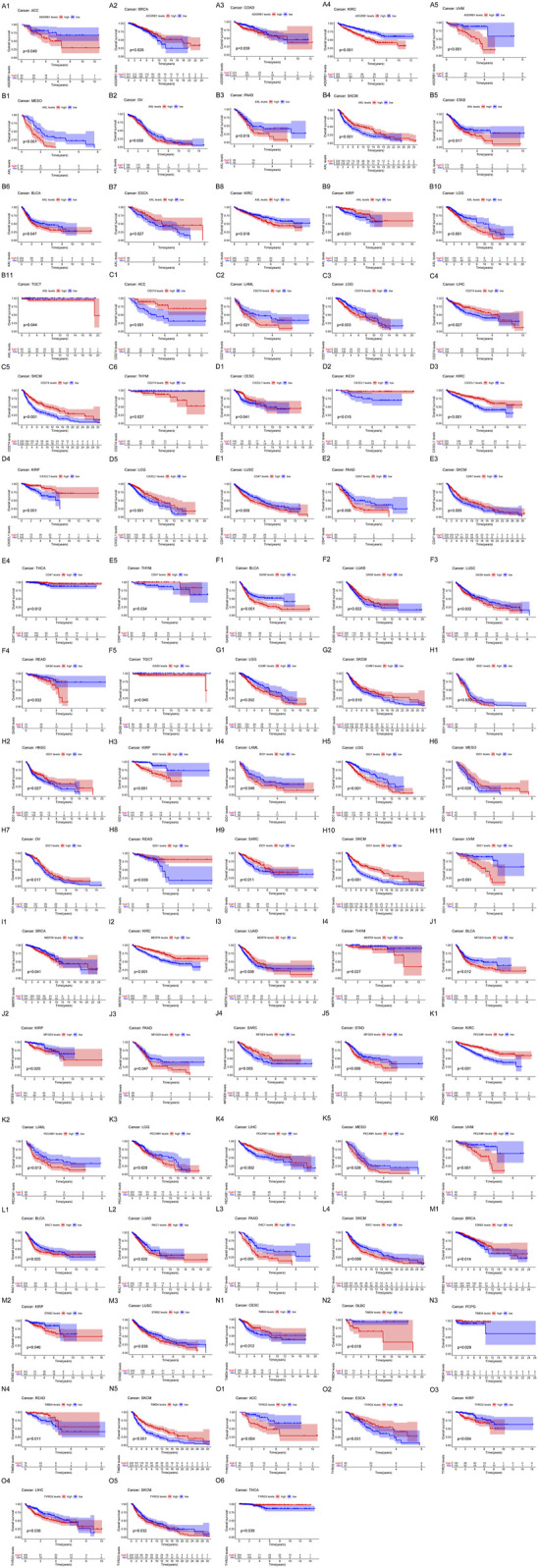
The correlation of the expression of efferocytosis-related molecules with the overall survival (OS) in 33 types of cancers. Kaplan-Meier survival curves were generated for the molecules extracted from the comparison of groups of high (red line) and low (blue line) gene expression. *p* < 0.05 in the log-rank test. **(A1–A5)** The expression of ADGRB1 (BAI1) was significantly associated with OS in ACC, BRCA, COAD, KIRC, and UVM. **(B1–B11)** The expression of Axl was significantly associated with OS in MESO, OV, PAAD, SKCM, STAD, BLCA, ESCA, KIRC, KIRP, LGG, and TGCT. **(C1–C6)** The expression of CD274 (PD-L1) was significantly associated with OS in ACC, LAML, LGG, LIHC, SKCM, and THYM. **(D1–D5)** The expression of CX3CL1 was significantly associated with OS in CESC, KICH, KIRC, KIRP, and LGG. **(E1–E5)** The expression of CD47 was significantly associated with OS in LUSC, PAAD, SKCM, THCA, and THYM. **(F1–F5)** The expression of Gas6 was significantly associated with OS in BLCA, LUAD, LUSC, READ, and TGCT. **(G1–G2)** The expression of ICAM1 was significantly associated with OS in LGG and SKCM. **(H1–H11)** The expression of IDO1 was significantly associated with OS in GBM, HNSC, KIRP, LAML, LGG, MESO, OV, READ, SARC, SKCM, and UVM. **(I1–I4)** The expression of MerTK was significantly associated with OS in BRCA, KIRC, LUAD, and THYM. **(J1–J5)** The expression of MFGE8 was significantly associated with OS in BLCA, KIRP, PAAD, SARC, and STAD. **(K1–K6)** The expression of PECAM1 (CD31) was significantly associated with OS in KIRC, LAML, LGG, LIHC, MESO, and UVM. **(L1–L4)** The expression of RAC1 was significantly associated with OS in BLCA, LUAD, PAAD, and SKCM. **(M1–M3)** The expression of STAB2 was significantly associated with OS in BRCA, KIRP, and LUSC. **(N1–N5)** The expression of TIMD4 was significantly associated with OS in CESC, DLBC, PCPG, READ, and SKCM. **(O1–O6)** The expression of Tyro3 was significantly associated with OS in ACC, ESCA, KIRP, LIHC, SKCM, and THCA.

We analyzed the expression of the 15 efferocytosis-related molecules in 16 types of cancers, including bladder urothelial carcinoma (BLCA), breast invasive carcinoma (BRCA), colon adenocarcinoma (COAD), esophageal carcinoma (ESCA), head and neck squamous cell carcinoma (HNSC), kidney chromophobe (KICH), kidney renal clear cell carcinoma (KIRC), kidney renal papillary cell carcinoma (KIRP), liver hepatocellular carcinoma (LIHC), lung adenocarcinoma (LUAD), lung squamous cell carcinoma (LUSC), prostate adenocarcinoma (PRAD), rectum adenocarcinoma (READ), stomach adenocarcinoma (STAD), thyroid carcinoma (THCA), and uterine corpus endometrial carcinoma (UCEC). The expression characteristics of the molecules in tumor and normal tissues in various cancers were different (Figure 2). In different cancers, the correlation between the expression of the molecules and OS was also different (Figure 3). CX3CL1 expression was increased in tumor tissues in KIRC and KIRP, predicting better OS. Axl expression was decreased in tumor tissues in BLCA, STAD and ESCA, predicting better OS in BLCA and STAD but worse OS in ESCA; Axl expression was increased in tumor tissues in KIRC and KIRP, predicting worse OS in KIRC but better OS in KIRP. Tyro3 expression was decreased in tumor tissues in ESCA and KIRP, predicting worse OS in ESCA but better OS in KIRP; Tyro3 expression was increased in tumor tissues in LIHC and THCA, predicting worse OS in LIHC but better OS in THCA. MerTK expression was decreased in tumor tissue in BRCA and KIRC, predicting better OS in BRCA but worse OS in KIRC. Gas6 expression was decreased in tumor tissues in BLCA, LUSC, and LUAD, predicting better OS in BLCA and LUSC but worse OS in LUAD. MFGE8 expression was decreased in tumor tissues in BLCA and KIRP, predicting better OS. BAI1 expression was decreased in tumor tissues in BRCA and COAD, predicting worse OS in BRCA but better OS in COAD. Tim-4 expression was decreased in tumor tissue in READ, predicting worse OS. Stab2 expression was decreased in tumor tissues in KIRP, LUSC, and BRCA, predicting better OS in KIRP and LUSC but worse OS in BRCA. CD31 expression was increased in tumor tissues in KIRC and LIHC, predicting better OS. CD47 expression was decreased in tumor tissue in LUSC, predicting better OS; CD47 expression was increased in tumor tissue in THCA, predicting better OS. IDO1 expression was increased in tumor tissues in KIRP and HNSC, predicting worse OS in KIRP but better OS in HNSC. PD-L1 expression was decreased in tumor tissue in LIHC, predicting worse OS. Based on the results, Axl may serve as new potential therapeutic targets for KIRC, as well as Tyro3 for LIHC and IDO1 for KIRP.

The expression of the 15 molecules was also significantly associated with other tumors. Although there was no differential expression between normal and tumor tissues, we should also pay attention to their association with other clinical classifications, such as tumor size, tumor stage, and tumor metastasis.

### Correlation with the tumor microenvironment

**FIGURE 4:**
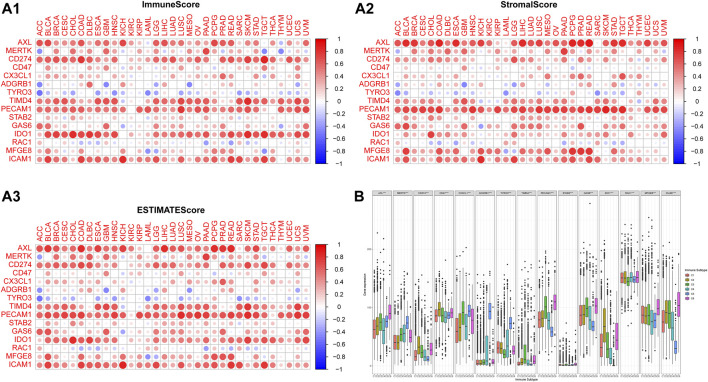
The correlations of the expressions of efferocytosis-related molecules with the microenvironment and immune subtype in 33 types of cancers. The corrplot package was used to assess the association of molecules with the immune score, stromal score, and ESTIMATE score. The color of a node reflects the log(FC) value of the Z score of gene expression, and the size of the node indicates the number of interacting proteins with the designated protein. **(A1)** The correlations of the expressions of efferocytosis-related molecules with the immune score. **(A2)** The correlations of the expressions of efferocytosis-related molecules with the stromal score. **(A3)** The correlations of the expressions of efferocytosis-related molecules with the ESTIMATE score. **(B)** Distribution of efferocytosis-related molecules in immune subtypes C1 (wound healing), C2 (IFN-γ-dominant), C3 (inflammatory), C4 (lymphocyte depleted), C5 (inflammatory) and C6 (TGF-β-dominant).

The high expressions of Axl, Tim-4, CD31, IDO1, ICAM1, and PD-L1 were associated with the high ESTIMATE score and immune score, while the high expressions of Axl, Tim-4, and CD31 were associated with the high stromal score in many types of cancers. High MerTK expression was associated with the high three scores in 3–5 types of cancers. The high expressions of Gas6 and MFGE8 were associated with the high ESTIMATE score and stromal score in certain types of cancers (Figure 4A).

According to a previous study, the C4 (lymphocyte-depleted) and C6 (TGF-β-dominant) subtypes were associated with poor OS (Thorsson et al., 2018). The expression levels of the 15 molecules in the C1-C6 subtypes were all significantly different. Tyro3, MerTK, and BAI1 were mainly expressed in the C5 (immunologically quiet) and C4 (lymphocyte-depleted) subtypes; Axl was mainly expressed in the C6 (TGF-β-dominant) and C5 (immunologically quiet) subtypes; CD31, Stab2, and Gas6 were mainly expressed in the C6 (TGF-β-dominant) and C3 (inflammatory) subtypes; CX3CL1 was mainly expressed in the C5 (immunologically quiet) and C3 (inflammatory) subtypes; Tim-4 was mainly expressed in the C3 (inflammatory) and C6 (TGF-β-dominant) subtypes; PD-L1, CD47, and IDO1 were mainly expressed in the C2 (IFN-γ-dominant) and C6 (TGF-β-dominant) subtypes; Rac1 was mainly expressed in the C6 (TGF-β-dominant) and C4 (lymphocyte-depleted) subtypes; MFGE8 was mainly expressed in the C6 (TGF-β-dominant) and C1 (low Th1/Th2 ratio) subtypes; ICAM1 was mainly expressed in the C6 (TGF-β-dominant) and C2 (IFN-γ-dominant) subtypes (Figure 4B).

### Correlation with anticancer sensitivity

**FIGURE 5:**
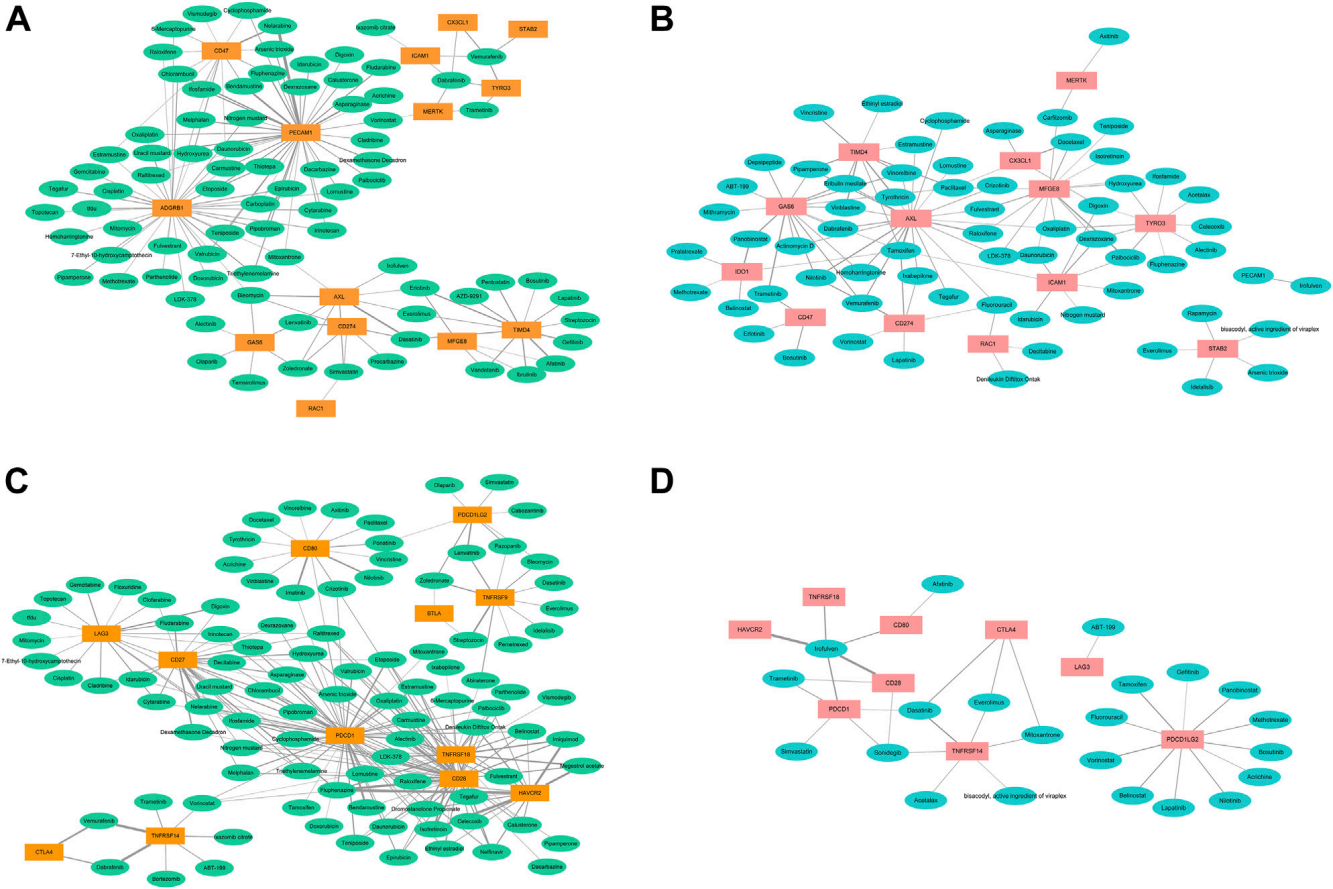
The correlations of the expressions of efferocytosis and immune checkpoint related molecules with sensitivity of the cancer cells to the 126 types of anti-cancer drugs. The thickness of the line indicates the size of the correlation coefficient. **(A)**. The correlations of the enhanced expressions of 14 efferocytosis-related molecules with increased sensitivity to anti-cancer drugs. **(B)** The correlations of the enhanced expresssions of 14 efferocytosis-related molecules with decreased sensitivity to anti-cancer drugs. **(C)** The correlations of the enhanced expressions of 12 immune checkpoint-related molecules with increased sensitivity to anti-cancer drugs. **(D)** The correlations of the enhanced expressions of 9 immune checkpoint-related molecules with decreased sensitivity to anti-cancer drugs.

We also analyzed the associations of the expressions of the 27 molecules with sensitivity of the cancer cells to the 126 types of anti-cancer drugs (Figure 5). Except for BAI1, CD31, and MerTK, the enhanced expression of Axl, Tyro3, Gas6, MFGE8, Stab2, Tim-4, CX3CL1, IDO1, Rac1, and PD-L1 was associated with decreased sensitivity to many drugs, which may partially explain the resistance to chemotherapy in cancers.

The enhanced expression of Axl, Tyro3, Gas6, MFGE8, Tim-4, and PD-L1 was associated with increased sensitivity to 22, 10, 16, 14, 10, and 6 types of drugs, respectively. With the correlation value greater than or equal to 0.400, enhanced expression of CX3CL1 was associated with increased sensitivity to vemurafenib but decreased sensitivity to docetaxel; enhanced expression of Axl was associated with increased sensitivity to bleomycin and dasatinib but decreased sensitivity to tamoxifen, nilotinib, and raloxifene; enhanced expression of Tyro3 was associated with increased sensitivity to vemurafenib; enhanced expression of Tim-4 was associated with increased sensitivity to dasatinib, vandetanib, pentostatin, ibrutinib, gefitinib, erlotinib, and afatinib but decreased sensitivity to vinblastine, tyrothricin, and pipamperone; enhanced expression of BAI1 was associated with increased sensitivity to etoposide, thiotepa, mitomycin, and cisplatin; enhanced expression of Gas6 was associated with decreased sensitivity to trametinib and panobinostat; enhanced expression of MFGE8 was associated with decreased sensitivity to palbociclib; enhanced expression of CD47 was associated with increased sensitivity to nelarabine; enhanced expression of CD31 was associated with increased sensitivity to etoposide, triethylenemelamine, thiotepa, dexamethasone decadron, fludarabine, melphalan, uracil mustard, pipobroman, ifosfamide, hydroxyurea, fluphenazine, chlorambucil, asparaginase, bendamustine, and nelarabine; enhanced expression of IDO1 was associated with decreased sensitivity to panobinostat; and enhanced expression of PD-L1 was associated with decreased sensitivity to tamoxifen.

For other immune checkpoint-related molecules, enhanced expressions of CD27, CD28, Tim-3, PD-1, and GITR were associated with increased sensitivity to many types of anticancer drugs, and only enhanced expressions of PD-1, CD28, CTLA4, and HVEM were associated with decreased sensitivity to a few drugs. In addition, enhanced expressions of Axl, PD-L1, and Rac1 were associated with decreased sensitivity to fluorouracil. Enhanced expressions of Axl, Gas6, and Tim-4 were associated with decreased sensitivity to pipamperone, eribulin mesylate, vinblastine and vinorelbine. Enhanced expressions of CX3CL1, Gas6, and Tim-4 were associated with decreased sensitivity to paclitaxel. Enhanced expressions of CTLA4, Stab2, and HVEM were associated with decreased sensitivity to everolimus. Enhanced expressions of CD28, CD80, Tim-3, PD-1, CD31, and GITR were associated with decreased sensitivity to irofulven. Enhanced expressions of Axl, PD-L1, Gas6, IDO1, and Tim-4 were associated with decreased sensitivity to tamoxifen. Enhanced expressions of CD28, CD47, Gas6, and PD-1 were associated with decreased sensitivity to trametinib. Enhanced expressions of CTLA4, PD-1, and HVEM were associated with decreased sensitivity to dasatinib. Enhanced expressions of Axl, PD-L1, and Gas6 were associated with decreased sensitivity to nilotinib. Enhanced expressions of CD28, PD-1, and HVEM were associated with decreased sensitivity to sonidegib.

The results indicate that we may choose the drugs according to the molecular expression characteristics and the correlation of their expression with anti-cancer drug sensitivity. For cancers with high expression of many types of molecules, we should choose drugs sensitive to the co-expression of the molecules or the combined use of the corresponding sensitive drugs.

## Discussion

The TAM receptors Axl, Tyro3, and MerTk were a family of receptor tyrosine kinases, skew macrophage polarization towards a pro-tumor M2-like phenotype in the tumor microenvironment, and promote apoptotic cell clearance through efferocytosis (Graham et al., 2014; Myers et al., 2019). The Gas6/Axl signaling pathway has been reported to be associated with tumor metastasis, invasion, and drug resistance (Zhu et al., 2019). Inhibition of the Gas6/Axl pathway augments the efficacy of chemotherapies (Kariolis et al., 2017; Wu et al., 2018). It was reported that Gas6 expression was associated with tumor progression and patient survival in RCC, and low tumor Axl mRNA levels were independently correlated with improved survival (Gustafsson et al., 2009). In the current study, Gas6 expression was decreased in KIRC tumor tissues, and Gas6 expression was decreased with tumor grades 1–3 and tumor metastasis. Axl was overexpressed in tumor tissues, and Axl overexpression predicted poor OS. These results were consistent with those from previous studies. Gas6/Axl axis contributes to chemoresistance and metastasis in breast cancer (Wang et al., 2016). Increased expression of Axl and its ligand Gas6 was found in EGFR-mutant lung cancers obtained from individuals with acquired resistance to erlotinib (Zhang et al., 2012). We found that Gas6 expression was decreased in tumor tissues in BLCA, LUSC, and LUAD, predicting a better OS in BLCA and LUSC but worse OS in LUAD, which indicated that the specific role of Gas6 in certain cancers needs to be further investigated. Furthermore, the drug sensitivity analyses showed that enhanced expression of Gas6 was moderately associated with increased sensitivity to alectinib, olaparib, temsirolimus, zoledronate, bleomycin, and lenvatinib, and we may also consider using these drugs to target Gas6. Different therapeutic agents targeting Axl have been developed (Zhu et al., 2019). We also found that high expression of Axl in tumor tissues was associated with worse OS in LGG, MESO, PAAD, and TGCT, and enhanced expression of Axl was especially associated with increased sensitivity to dasatinib and bleomycin. We may consider using these drugs to treat the above cancers.

Tyro3, Axl, and MerTK control myeloid-derived suppressor cell (MDSC) functionality, regulate MDSC-mediated immune suppression and augment anti-PD-1 therapy in melanoma patients (Holtzhausen et al., 2019), which highlights the role of these molecules in antitumor therapy. In leiomyosarcoma patients, especially those whom develop metastasis, express higher levels of Tyro3 and Gas6, and crizotinib and foretinib showed effective antitumor activity in leiomyosarcoma through Tyro3 and Axl deactivation (Dantas-Barbosa et al., 2017). Based on our study, Tyro3 expression was increased in tumor tissue in LIHC, predicting worse OS. Tyro3 was reported to contribute significantly to tumor growth, aggressiveness and liver dysfunction in HCC (Duan et al., 2016) and was also considered a marker and therapeutic target for HCC with a higher hepatitis activity (Tsai et al., 2020). Sorafenib is the only approved drug for treating patients with advanced HCC. However, the therapeutic effect of sorafenib is transient, and patients invariably develop sorafenib resistance through the aberrant expression of the Tyro3/phosphoinositide 3-kinase/protein kinase B signal transduction pathway (Kabir et al., 2018). We found that enhanced expression of Tyro3 was associated with increased sensitivity to trametinib, dabrafenib, and vemurafenib, and we may consider using these drugs to target Tyro3 in HCC.

MFGE8 acts at two levels, by increasing vascular endothelial growth factor (VEGF) and ET-1 expression in mesenchymal stromal cells and by enhancing M2 polarization of macrophages, to increase melanoma tumor angiogenesis (Yamada et al., 2016). The abundant MFGE8 expression in esophageal squamous cell carcinoma might have a negative influence on the long-term survival of patients after chemotherapy (Kanemura et al., 2018). MFGE8 also plays an important role in HCC progression (Ko et al., 2020). Our results showed that high expression of MFGE8 in tumor tissues was also associated with worse OS in PAAD and STAD, which indicated that MFGE8 may serve as a potential target in these cancers.

BAI1 expression was significantly reduced in breast cancer and higher expression was associated with better patient survival (Meisen et al., 2015). Our results were consistent with those from previous studies. BAI1 is decreased in BRCA and associated with worse OS. It was reported that BAI1 had better diagnostic efficacy than classic lung cancer biomarkers (Zhang et al., 2019). Blocking Stab2 function prevents melanoma metastasis by elevating circulating hyaluronic acid levels (Hirose et al., 2012). In our analysis, Stab2 expression was decreased in tumor tissues in KIRP, LUSC, and BRCA, predicting better OS in KIRP and LUSC but worse OS in BRCA, which indicated that the specific role of Stab2 in different cancers needs to be further investigated.

Tim-4-overexpressing cancer cells recruit TAMs, thereby accelerating cancer development. Tim-4 promotes the growth of colorectal cancer by activating angiogenesis and recruiting TAMs *via* the PI3K/AKT/mTOR signaling pathway (Tan et al., 2018). Tim-4 was also reported to promote the growth of NSCLC in an RGD motif-dependent manner (Zhang et al., 2015). In addition, IL-6 promotes metastasis of NSCLC by upregulating Tim-4 *via* NF-κB (Liu et al., 2020). In the current study, the expression level of Tim-4 in tumor tissues was significantly higher in KICH, KIRC, KIRP, and UCEC, and the specific role of Tim-4 in these cancers needs further investigation. Interestingly, Tim-3 is overexpressed in RCC tumor tissues, and overexpression of Tim-3 predicts better DFS and OS and has no significant correlation with the TNM classification and tumor grade (data not shown); this may partially explain the results that dual blockade of PD-1 and LAG-3, but not PD-1 and Tim-3, is a promising checkpoint blockade combination strategy (Zelba et al., 2019).

Rac1 plays a critical role in the progression of tumors and the development of resistance to various therapeutic modalities applied in the clinic (De et al., 2020). The overexpression of Rac1 is associated with multi-drug resistance to the neoadjuvant chemotherapy, and targeting Rac1 is a potential strategy to overcome acquired chemoresistance in breast cancer (Li et al., 2020). We found that the expression level of Rac1 in tumor tissues was significantly higher in BRCA, KICH, KIRP, and THCA, and we should also pay attention to the role of Rac1 in KICH, KIRP, and THCA.

CD47 modulates cellular phagocytosis by macrophages, transmigration of neutrophils and activation of dendritic cells, T cells and B cells. Anti-CD47 antibodies, which enhance cancer cell phagocytosis, can achieve higher anti-cancer efficacies when combined with chemotherapy and immunotherapy (Hayat et al., 2020). Our study showed that COAD, HNSC, KICH, STAD, and THCA tumor tissues expressed high levels of CD47, and high expression of CD47 in tumor tissue was associated with worse OS in PAAD. Based on this observation, CD47 may be considered a target in the above cancer therapies.

IDO1 protein is expressed in the majority of hormone receptor-positive breast cancer and is an independent negative prognostic marker (Carvajal-Hausdorf et al., 2017). IDO1 expression was associated with an unfavorable clinical outcome in esophageal cancer and colorectal cancer (Kiyozumi et al., 2019; Chen et al., 2020). IDO1 expression was also reported to correlate with PD-L1 expression, and co-expressed IDO1 and PD-L1 may be an important target for immunotherapy in lung squamous cell carcinoma (Takada et al., 2019). In the current study, IDO1 expression was increased in tumor tissue in KIRP, predicting worse OS, which suggested that IDO1 may serve as a potential target for KIRP. IDO1, LAG3, and PD-L1 expression levels in TIICs showed a better prognosis for patients with MSI-H colon cancer (Lee et al., 2018). Thus, the potential therapeutic implications of these immune checkpoint molecules should be further investigated.

PD-1 and PD-L1 are generally considered as potential targets for antitumor therapy (Balar and Weber, 2017; Constantinidou et al., 2019). Clinical trials with mAbs to PD-1 and PD-L1 have shown impressive response rates in patients, particularly for melanoma, NSCLC, RCC, and bladder cancer (Ohaegbulam et al., 2015). In a multicenter phase 1 trial, antibody-mediated blockade of PD-L1 induced durable tumor regression and prolonged stabilization of disease in patients with advanced cancers (Brahmer et al., 2012). Cancer vaccine formulation can dominantly determine synergy, or lack thereof, with CTLA-4 and PD-L1 checkpoint blockade therapy for cancer (Hailemichael et al., 2018). Based on our results, enhanced expression of PD-1 was associated with increased sensitivity to many types of drugs, and enhanced expression of CTLA4 was associated with increased sensitivity to dabrafenib and vemurafenib; however, enhanced expression of PD-L1 was associated with decreased sensitivity to vorinostat, tamoxifen, nilotinib, ixabepilone, lapatinib, and fluorouracil. In clinical use, we should pay more attention to the use of these drugs in patients with a high expression of these molecules.

The limitations of the study should be noted. Firstly, the results of our study were based on online database, and the patient number of some types of cancers was small, especially the number of normal control. We also tried to search other database to confirm our results, such as GEO DataSets (https://www.ncbi.nlm.nih.gov/geo/). However, no survival time data were available. The results need further confirmation both in clinic and experimental model. Secondly, there are data to suggest that some of the efferocytosis-related receptors are important in hematologic cancers and may have an interface with the immune checkpoint molecules in the bone marrow microenvironment. We did not perform the analysis on hematologic malignancies in the current study. Finally, not all efferocytosis-related molecular were included in, such as other TAM receptors ligands Protein S and Galectin 3, and alpha-v/beta-5 receptor.

In summary, our results provided evidence that efferocytosis-related molecules played important roles in the invasion, metastasis, and clinical outcome of many types of cancers, and their correlation was different in each cancer. The high expression of the molecules was associated with the high ESTIMATE score, stromal score, and immune score. Except for CX3CL1, the other 14 molecules were mainly expressed in the C4 (lymphocyte-depleted) and C6 (TGF-β-dominant) subtypes, which predicted worse OS. The molecules also played important roles in resistance to chemotherapy. We may choose anticancer drugs according to the molecular expression characteristics in each tumor and the drug sensitivity results. Supplementing conventional chemotherapy, radiotherapy and other immunotherapies with efferocytosis-targeted therapy could enhance therapeutic efficacy, reduce resistance to therapy, and promote patient outcome.

## Data Availability

Publicly available datasets were analyzed in this study. These data can be found in The Cancer Genome Atlas (http://xena.ucsc.edu/), the CellMiner database v2.2 (https://discover.nci.nih.gov/cellminer/), and the GEPIA2 website (http://gepia2.cancer-pku.cn/#index).

## References

[B1] Balar A. V. Weber J. S. (2017). PD-1 and PD-L1 antibodies in cancer: Current status and future directions. Cancer Immunol. Immunother. 66 (5), 551–564. 10.1007/s00262-017-1954-6 28213726 PMC11028560

[B2] Bid H. K. Roberts R. D. Manchanda P. K. Houghton P. J. (2013). RAC1: An emerging therapeutic option for targeting cancer angiogenesis and metastasis. Mol. Cancer Ther. 12 (10), 1925–1934. 10.1158/1535-7163.MCT-13-0164 24072884 PMC3823055

[B3] Brahmer J. R. Tykodi S. S. Chow L. Q. Hwu W. J. Topalian S. L. Hwu P. (2012). Safety and activity of anti-PD-L1 antibody in patients with advanced cancer. N. Engl. J. Med. 366 (26), 2455–2465. 10.1056/NEJMoa1200694 22658128 PMC3563263

[B4] Carvajal-Hausdorf D. E. Mani N. Velcheti V. Schalper K. A. Rimm D. L. (2017). Objective measurement and clinical significance of Ido1 protein in hormone receptor-positive breast cancer. J. Immunother. Cancer 5 (1), 81. 10.1186/s40425-017-0285-7 29037255 PMC5644103

[B5] Chen B. Alvarado D. M. Iticovici M. Kau N. S. Park H. Parikh P. J. (2020). Interferon-induced Ido1 mediates radiation resistance and is a therapeutic target in colorectal cancer. Cancer Immunol. Res. 8 (4), 451–464. 10.1158/2326-6066.CIR-19-0282 32127391 PMC7123802

[B6] Constantinidou A. Alifieris C. Trafalis D. T. (2019). Targeting programmed cell death -1 (PD-1) and ligand (PD-L1): A new era in cancer active immunotherapy. Pharmacol. Ther. 194, 84–106. 10.1016/j.pharmthera.2018.09.008 30268773

[B7] Dantas-Barbosa C. Lesluyes T. Loarer F. L. Chibon F. Treilleux I. Coindre J. M. (2017). Expression and role of TYRO3 and AXL as potential therapeutical targets in leiomyosarcoma. Br. J. Cancer 117 (12), 1787–1797. 10.1038/bjc.2017.354 29024938 PMC5729471

[B8] Datar I. Sanmamed M. F. Wang J. Henick B. S. Choi J. Badri T. (2019). Expression analysis and significance of PD-1, LAG-3, and TIM-3 in human non-small cell lung cancer using spatially resolved and multiparametric single-cell analysis. Clin. Cancer Res. 25 (15), 4663–4673. 10.1158/1078-0432.CCR-18-4142 31053602 PMC7444693

[B9] De P. Rozeboom B. J. Aske J. C. Dey N. (2020). Active RAC1 promotes tumorigenic phenotypes and therapy resistance in solid tumors. Cancers (Basel) 12 (6), 1541. 10.3390/cancers12061541 PMC7352592 32545340

[B10] Duan Y. Wong W. Chua S. C. Wee H. L. Lim S. G. Chua B. T. (2016). Overexpression of Tyro3 and its implications on hepatocellular carcinoma progression. Int. J. Oncol. 48 (1), 358–366. 10.3892/ijo.2015.3244 26573872

[B11] Galon J. Bruni D. (2019). Approaches to treat immune hot, altered and cold tumours with combination immunotherapies. Nat. Rev. Drug Discov. 18 (3), 197–218. 10.1038/s41573-018-0007-y 30610226

[B12] Graham D. K. DeRyckere D. Davies K. D. Earp H. S. (2014). The TAM family: Phosphatidylserine sensing receptor tyrosine kinases gone awry in cancer. Nat. Rev. Cancer 14 (12), 769–785. 10.1038/nrc3847 25568918

[B13] Gustafsson A. Martuszewska D. Johansson M. Ekman C. Hafizi S. Ljungberg B. (2009). Differential expression of Axl and Gas6 in renal cell carcinoma reflecting tumor advancement and survival. Clin. Cancer Res. 15 (14), 4742–4749. 10.1158/1078-0432.CCR-08-2514 19567592

[B14] Hailemichael Y. Woods A. Fu T. He Q. Nielsen M. C. Hasan F. (2018). Cancer vaccine formulation dictates synergy with CTLA-4 and PD-L1 checkpoint blockade therapy. J. Clin. Invest. 128 (4), 1338–1354. 10.1172/JCI93303 29480817 PMC5873868

[B15] Hayat S. M. G. Bianconi V. Pirro M. Jaafari M. R. Hatamipour M. Sahebkar A. (2020). CD47: Role in the immune system and application to cancer therapy. Cell. Oncol. 43 (1), 19–30. 10.1007/s13402-019-00469-5 PMC12990683 31485984

[B16] Hirose Y. Saijou E. Sugano Y. Takeshita F. Nishimura S. Nonaka H. (2012). Inhibition of Stabilin-2 elevates circulating hyaluronic acid levels and prevents tumor metastasis. Proc. Natl. Acad. Sci. U. S. A. 109 (11), 4263–4268. 10.1073/pnas.1117560109 22371575 PMC3306694

[B17] Holtzhausen A. Harris W. Ubil E. Hunter D. M. Zhao J. Zhang Y. (2019). TAM family receptor kinase inhibition reverses MDSC-mediated suppression and augments anti-PD-1 therapy in melanoma. Cancer Immunol. Res. 7 (10), 1672–1686. 10.1158/2326-6066.CIR-19-0008 31451482 PMC6943983

[B18] Kabir T. D. Ganda C. Brown R. M. Beveridge D. J. Richardson K. L. Chaturvedi V. (2018). A microRNA-7/growth arrest specific 6/TYRO3 axis regulates the growth and invasiveness of sorafenib-resistant cells in human hepatocellular carcinoma. Hepatology 67 (1), 216–231. 10.1002/hep.29478 28833396

[B19] Kanemura T. Miyata H. Makino T. Tanaka K. Sugimura K. Hamada-Uematsu M. (2018). Immunoregulatory influence of abundant MFG-E8 expression by esophageal cancer treated with chemotherapy. Cancer Sci. 109 (11), 3393–3402. 10.1111/cas.13785 30156356 PMC6215892

[B20] Kariolis M. S. Miao Y. R. Diep A. Nash S. E. Olcina M. M. Jiang D. (2017). Inhibition of the GAS6/AXL pathway augments the efficacy of chemotherapies. J. Clin. Invest. 127 (1), 183–198. 10.1172/JCI85610 27893463 PMC5199716

[B21] Kiyozumi Y. Baba Y. Okadome K. Yagi T. Ishimoto T. Iwatsuki M. (2019). Ido1 expression is associated with immune tolerance and poor prognosis in patients with surgically resected esophageal cancer. Ann. Surg. 269 (6), 1101–1108. 10.1097/SLA.0000000000002754 31082908

[B22] Ko D. S. Kim S. H. Park J. Y. Lee G. Kim H. J. Kim G. (2020). Milk fat globule-EGF factor 8 contributes to progression of hepatocellular carcinoma. Cancers (Basel) 12 (2), 403. 10.3390/cancers12020403 PMC7072366 32050643

[B23] Lee S. J. Jun S. Y. Lee I. H. Kang B. W. Park S. Y. Kim H. J. (2018). CD274, LAG3, and Ido1 expressions in tumor-infiltrating immune cells as prognostic biomarker for patients with MSI-high colon cancer. J. Cancer Res. Clin. Oncol. 144 (6), 1005–1014. 10.1007/s00432-018-2620-x 29520442 PMC11813403

[B24] Li Q. Qin T. Bi Z. Hong H. Ding L. Chen J. (2020). Rac1 activates non-oxidative pentose phosphate pathway to induce chemoresistance of breast cancer. Nat. Commun. 11 (1), 1456. 10.1038/s41467-020-15308-7 32193458 PMC7081201

[B25] Liu W. Wang H. Bai F. Ding L. Huang Y. Lu C. (2020). IL-6 promotes metastasis of non-small-cell lung cancer by up-regulating TIM-4 via NF-κB. Cell Prolif. 53 (3), e12776. 10.1111/cpr.12776 32020709 PMC7106962

[B26] Meisen W. H. Dubin S. Sizemore S. T. Mathsyaraja H. Thies K. Lehman N. L. (2015). Changes in Bai1 and nestin expression are prognostic indicators for survival and metastases in breast cancer and provide opportunities for dual targeted therapies. Mol. Cancer Ther. 14 (1), 307–314. 10.1158/1535-7163.MCT-14-0659 25376607 PMC4297221

[B27] Morioka S. Maueroder C. Ravichandran K. S. (2019). Living on the edge: Efferocytosis at the interface of homeostasis and pathology. Immunity 50 (5), 1149–1162. 10.1016/j.immuni.2019.04.018 31117011 PMC6721617

[B28] Motzer R. J. Tannir N. M. McDermott D. F. Aren Frontera O. Melichar B. Choueiri T. K. (2018). Nivolumab plus ipilimumab versus sunitinib in advanced renal-cell carcinoma. N. Engl. J. Med. 378 (14), 1277–1290. 10.1056/NEJMoa1712126 29562145 PMC5972549

[B29] Myers K. V. Amend S. R. Pienta K. J. (2019). Targeting Tyro3, Axl and MerTK (TAM receptors): Implications for macrophages in the tumor microenvironment. Mol. Cancer 18 (1), 94. 10.1186/s12943-019-1022-2 31088471 PMC6515593

[B30] Ohaegbulam K. C. Assal A. Lazar-Molnar E. Yao Y. Zang X. (2015). Human cancer immunotherapy with antibodies to the PD-1 and PD-L1 pathway. Trends Mol. Med. 21 (1), 24–33. 10.1016/j.molmed.2014.10.009 25440090 PMC4282825

[B31] Pacheco J. M. (2020). KEYNOTE-407: Changing the way we treat stage IV squamous non-small cell lung cancer. Transl. Lung Cancer Res. 9 (1), 148–153. 10.21037/tlcr.2020.01.12 32206562 PMC7082291

[B32] Proto J. D. Doran A. C. Gusarova G. Yurdagul A. Jr. Sozen E. Subramanian M. (2018). Regulatory T cells promote macrophage efferocytosis during inflammation resolution. Immunity 49 (4), 666–677. 10.1016/j.immuni.2018.07.015 30291029 PMC6192849

[B33] Takada K. Kohashi K. Shimokawa M. Haro A. Osoegawa A. Tagawa T. (2019). Co-expression of Ido1 and PD-L1 in lung squamous cell carcinoma: Potential targets of novel combination therapy. Lung Cancer 128, 26–32. 10.1016/j.lungcan.2018.12.008 30642449

[B34] Tan X. Zhang Z. Yao H. Shen L. (2018). Tim-4 promotes the growth of colorectal cancer by activating angiogenesis and recruiting tumor-associated macrophages via the PI3K/AKT/mTOR signaling pathway. Cancer Lett. 436, 119–128. 10.1016/j.canlet.2018.08.012 30118845

[B35] Thorsson V. Gibbs D. L. Brown S. D. Wolf D. Bortone D. S. Ou Yang T. H. (2018). The immune landscape of cancer. Immunity 48 (4), 812–830. e814. 10.1016/j.immuni.2018.03.023 29628290 PMC5982584

[B36] Tsai C. L. Chang J. S. Yu M. C. Lee C. H. Chen T. C. Chuang W. Y. (2020). Functional Genomics identifies hepatitis-induced STAT3-TYRO3-STAT3 signaling as a potential therapeutic target of hepatoma. Clin. Cancer Res. 26 (5), 1185–1197. 10.1158/1078-0432.CCR-18-3531 31831556

[B37] Usami Y. Ishida K. Sato S. Kishino M. Kiryu M. Ogawa Y. (2013). Intercellular adhesion molecule-1 (ICAM-1) expression correlates with oral cancer progression and induces macrophage/cancer cell adhesion. Int. J. Cancer 133 (3), 568–578. 10.1002/ijc.28066 23364881

[B38] Wang C. Jin H. Wang N. Fan S. Wang Y. Zhang Y. (2016). Gas6/Axl Axis contributes to chemoresistance and metastasis in breast cancer through akt/GSK-3β/β-catenin signaling. Theranostics 6 (8), 1205–1219. 10.7150/thno.15083 27279912 PMC4893646

[B39] Wang Q. Zhang J. Tu H. Liang D. Chang D. W. Ye Y. (2019). Soluble immune checkpoint-related proteins as predictors of tumor recurrence, survival, and T cell phenotypes in clear cell renal cell carcinoma patients. J. Immunother. Cancer 7 (1), 334. 10.1186/s40425-019-0810-y 31783776 PMC6884764

[B40] Werfel T. A. Cook R. S. (2018). Efferocytosis in the tumor microenvironment. Semin. Immunopathol. 40 (6), 545–554. 10.1007/s00281-018-0698-5 30187085 PMC6223858

[B41] Werfel T. A. Elion D. L. Rahman B. Hicks D. J. Sanchez V. Gonzales-Ericsson P. I. (2019). Treatment-induced tumor cell apoptosis and secondary necrosis drive tumor progression in the residual tumor microenvironment through MerTK and Ido1. Cancer Res. 79 (1), 171–182. 10.1158/0008-5472.CAN-18-1106 30413412

[B42] Wu G. Ma Z. Cheng Y. Hu W. Deng C. Jiang S. (2018). Targeting Gas6/TAM in cancer cells and tumor microenvironment. Mol. Cancer 17 (1), 20. 10.1186/s12943-018-0769-1 29386018 PMC5793417

[B43] Yamada K. Uchiyama A. Uehara A. Perera B. Ogino S. Yokoyama Y. (2016). MFG-E8 drives melanoma growth by stimulating mesenchymal stromal cell-induced angiogenesis and M2 polarization of tumor-associated macrophages. Cancer Res. 76 (14), 4283–4292. 10.1158/0008-5472.CAN-15-2812 27197197 PMC5033700

[B44] Zelba H. Bedke J. Hennenlotter J. Mostbock S. Zettl M. Zichner T. (2019). PD-1 and LAG-3 dominate checkpoint receptor-mediated T-cell inhibition in renal cell carcinoma. Cancer Immunol. Res. 7 (11), 1891–1899. 10.1158/2326-6066.CIR-19-0146 31484656

[B45] Zhang L. Pu D. Liu D. Wang Y. Luo W. Tang H. (2019). Identification and validation of novel circulating biomarkers for early diagnosis of lung cancer. Lung Cancer 135, 130–137. 10.1016/j.lungcan.2019.06.019 31446985

[B46] Zhang Q. Wang H. Wu X. Liu B. Liu W. Wang R. (2015). TIM-4 promotes the growth of non-small-cell lung cancer in a RGD motif-dependent manner. Br. J. Cancer 113 (10), 1484–1492. 10.1038/bjc.2015.323 26512878 PMC4815884

[B47] Zhang Z. Lee J. C. Lin L. Olivas V. Au V. LaFramboise T. (2012). Activation of the AXL kinase causes resistance to EGFR-targeted therapy in lung cancer. Nat. Genet. 44 (8), 852–860. 10.1038/ng.2330 22751098 PMC3408577

[B48] Zhou Y. Yao Y. Deng Y. Shao A. (2020). Regulation of efferocytosis as a novel cancer therapy. Cell Commun. Signal. 18 (1), 71. 10.1186/s12964-020-00542-9 32370748 PMC7199874

[B49] Zhu C. Wei Y. Wei X. (2019). AXL receptor tyrosine kinase as a promising anti-cancer approach: Functions, molecular mechanisms and clinical applications. Mol. Cancer 18 (1), 153. 10.1186/s12943-019-1090-3 31684958 PMC6827209

